# A new species of *Wuliphantes* from Sichuan, China, with re-description on the type specimens of *W.tongluensis* (Araneae, Linyphiidae)

**DOI:** 10.3897/BDJ.11.e114390

**Published:** 2023-12-20

**Authors:** Lan Yang, Zhiyuan Yao, Shuqiang Li

**Affiliations:** 1 College of Life Science, Shenyang Normal University, Shenyang, China College of Life Science, Shenyang Normal University Shenyang China; 2 Institute of Zoology, Chinese Academy of Sciences, Beijing, China Institute of Zoology, Chinese Academy of Sciences Beijing China

**Keywords:** Asia, biodiversity, linyphiid spider, morphology, taxonomy

## Abstract

**Background:**

The genus *Wuliphantes* Irfan, Wang & Zhang, 2023 is a small genus in the family Linyphiidae Blackwall, 1859, with only three species: *W.guanshan* (Irfan, Wang & Zhang, 2022), *W.tongluensis* (Chen & Song, 1988) and *W.trigyrus* Irfan, Wang & Zhang, 2023, all distributed in China.

**New information:**

A new species: *Wuliphantesyaan* sp. nov. from Sichuan Province, China is reported. In addition, we re-described the type specimens of *W.tongluensis* (Chen & Song, 1988) that is similar to *W.yaan* sp. nov.

## Introduction

Linyphiidae Blackwall,1859 is the second most diverse spider family in Araneae and is widely distributed in the world, comprising 4845 species belonging to 634 genera (World Spider Catalog 2023). About 524 species in 180 genera are distributed in China and, amongst them, 46 species and 22 genera are distributed in Sichuan Province (Li 2020, Irfan et al. 2022, World Spider Catalog 2023, Yang et al. 2023).

The genus *Wuliphantes* is endemic to China (World Spider Catalog 2023). This genus can be distinguished from other genera in Linyphiidae by the following characteristics: long ventral projection of embolic plate; embolus clockwise, with more than one coil; transparent copulatory ducts; spiral-shaped spermathecae and scape and paramula absent (Irfan et al. 2023). In this paper, the type specimens of *W.tongluensis* is re-described and a new species *W.yaan* sp. nov. is reported.

## Materials and methods

Specimens were examined and measured with a Leica M205 C stereomicroscope. Left male palps were photographed. Epigynes were photographed before dissection. Vulvae were treated in a solution of trypsin enzyme to dissolve soft tissues before photography. Images were captured with an Olympus C7070 zoom digital camera (7.1 megapixels) and assembled using Helicon Focus 6.7.1 image stacking software (Khmelik et al. 2005). All measurements are given in millimetres (mm). Leg measurements are shown as: total length (femur, patella, tibia, metatarsus, tarsus). Leg segments were measured on their dorsal side. The metatarsal trichobothrium (Tm) value is given as the ratio of the distance between the proximal margin of the metatarsus and the root of the trichobothrium divided by the total length of the metatarsus and the Tm value for the first and the fourth leg is given as TmI and TmIV, respectively (Denis 1949, Locket and Millidge 1953, Zhao and Li 2014). The specimens studied are preserved in 75% ethanol and deposited in the Institute of Zoology, Chinese Academy of Sciences (IZCAS) in Beijing, China. Terminology and taxonomic descriptions follow Irfan et al. (2023).

The abbreviations are used in the text and figures: ALE = anterior lateral eye, AME = anterior median eye, AME–ALE = the distance between AME and ALE, AME–AME = the distance between AMEs, CD = copulatory duct, CO = copulatory opening, DP = dorsal plate, E = embolus, EPL = embolic plate, FD = fertilisation duct, MM = median membrane, PC = paracymbium, PLE = posterior lateral eye, PME = posterior median eye, PME–PLE = the distance between PME and PLE, PME–PME = the distance between PMEs, PT = protegulum, S = spermatheca, ST = subtegulum, T = tegulum, VP = ventral plate, VPE = ventral projection of embolic plate.

## Taxon treatments

### Wuliphantes tongluensis

(Chen & Song, 1988)

9345F1D1-4DB0-5BAB-96D2-B68566D72835

urn:lsid:nmbe.ch:spidersp:009601

#### Materials

- **Type status:** Holotype. **Occurrence:** recordedBy: Zhangfu Chen; individualCount: 1; sex: female; lifeStage: adult; occurrenceID: A9FF000B-0D6A-5E86-A968-908141E179EB; **Taxon:** order: Araneae; family: Linyphiidae; genus: Wuliphantes; **Location:** country: China; stateProvince: Zhejiang; municipality: Hangzhou; locality: Tonglu County; **Event:** year: 1985; month: 6; day: 6; **Record Level:** institutionCode: IZCAS-Ar 10017
- **Type status:** Allotype. **Occurrence:** recordedBy: Zhangfu Chen; individualCount: 1; sex: male; lifeStage: adult; occurrenceID: 4B1CA516-2BA3-53EB-9A50-E1E8EE6D7CAC; **Taxon:** order: Araneae; family: Linyphiidae; genus: Wuliphantes; **Location:** country: China; stateProvince: Zhejiang; municipality: Hangzhou; locality: Tonglu County; **Event:** year: 1985; month: 6; day: 6; **Record Level:** institutionCode: IZCAS-Ar 10018
- **Type status:** Other material. **Occurrence:** recordedBy: Lihong Tu, Shuqiang Li; individualCount: 1; sex: male; lifeStage: adult; occurrenceID: 1C4FDE70-E845-5D57-A51D-0A6E548A22F2; **Taxon:** order: Araneae; family: Linyphiidae; genus: Wuliphantes; **Location:** country: China; stateProvince: Hunan; municipality: Hengshan; locality: Nanyue District; verbatimLocality: Nanyue Hengshan Scenic Area; **Event:** year: 2003; month: 10; day: 11; **Record Level:** institutionCode: IZCAS

#### Description

For measurements data of *Wuliphantestongluensis*, see Chen and Song (1988).

**Figure 1.**
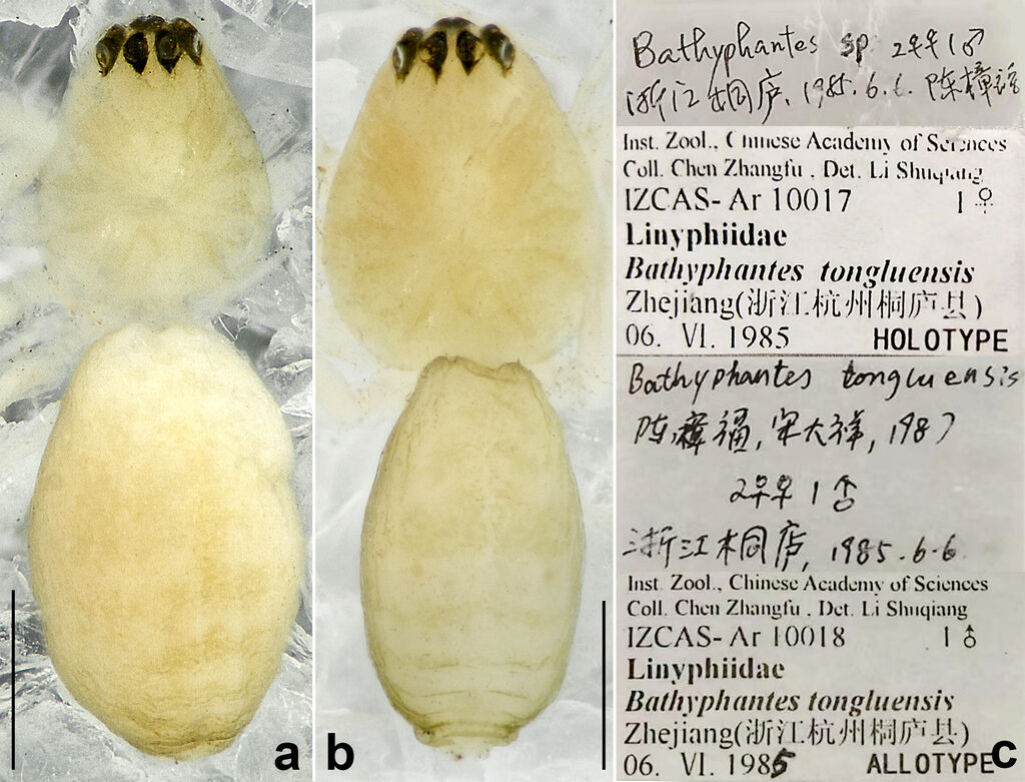
*Wuliphantestongluensis*, habitus of female holotype and male allotype, dorsal view (a, b) and original labels (c). **a** female; **b** male; **c** original labels. Scale bars: 0.50 mm (a, b).

**Female** (Holotype). Colour faded (Fig. 1a). Epigyne (Fig. 2a). Ventral plate wider than long; copulatory openings apparent, present posteriorly; dorsal plate inverted triangle; copulatory ducts forming five loops; spermathecae spiral; fertilisation ducts pointing antero-medially.

**Figure 3.**
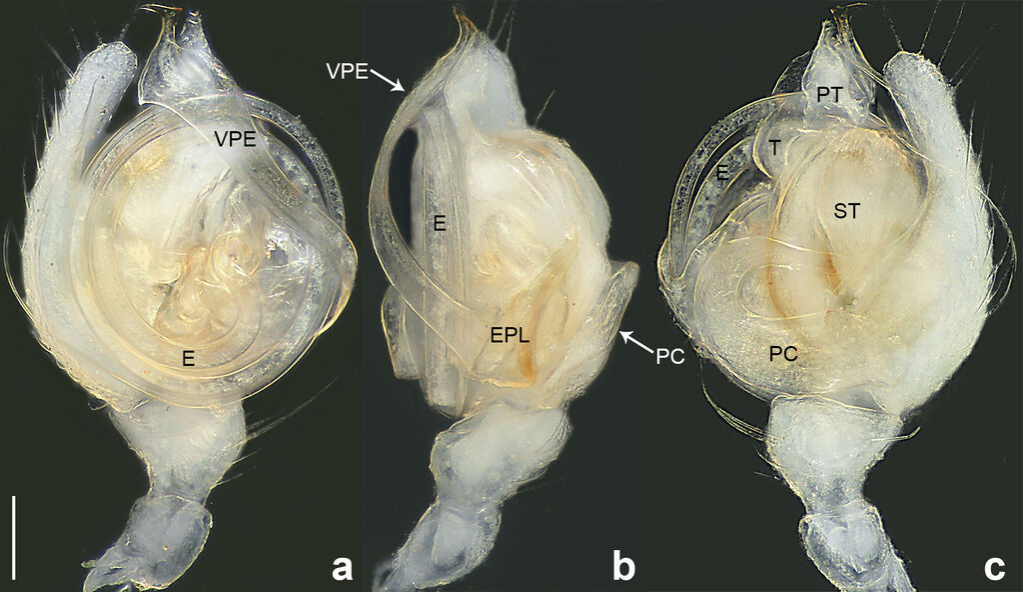
*Wuliphantestongluensis*, allotype male, left palp. **a** prolateral view; **b** ventral view; **c** retrolateral view. Scale bar: 0.10 mm (a–c).

**Male** (Allotype). Colour faded (Fig. 1b). Palp (Figs 3, 5a). Tibia conical; paracymbium U-shaped in retrolateral view, with wide base and narrow end; tegulum semicircle, with subtriangular protegulum; embolic plate well-developed, with ribbon-like ventral projection of embolic plate, the narrow distal protrusion faces right on the retrolateral view; embolus spiral, with two and a half coils, originating at 6 o’clock position in prolateral view.

#### Diagnosis

The species resembles *Wuliphantesyaan* sp. nov. with similar paracymbium and subtegulum, but can be distinguished by the embolus forming 2.5 coils (Figs 3, 5a; 1.5 coils in *W.yaan* sp. nov.); by the width of ventral projection of embolic plate increasing distally (Fig. 3a; width almost consistent in *W.yaan* sp. nov.); by the ventral projection of embolic plate with sharp and narrow distal protrusion (Fig. 3a; hooked in *W.yaan* sp. nov.); and by the copulatory ducts with five loops (Fig. 2a; two loops in *W.yaan* sp. nov.).

#### Distribution

China (Zhejiang, type locality; Anhui; Chongqing; Hubei; Hunan).

### Wuliphantes yaan

Yao & Li sp. nov.

BBF8F1B7-35FE-507B-8FF9-09E2B905F790

FF9ABE37-2A3B-43CC-9306-E58DD4B8B9EC

#### Materials

- **Type status:** Holotype. **Occurrence:** recordedBy: Lihong Tu; individualCount: 1; sex: male; lifeStage: adult; occurrenceID: 1FDFF9D1-30ED-5862-8799-440BE5DA8A8E; **Taxon:** order: Araneae; family: Linyphiidae; genus: Wuliphantes; **Location:** country: China; stateProvince: Sichuan; municipality: Ya'an; locality: Lushan County; **Event:** year: 2004; month: 7; day: 7; **Record Level:** institutionID: IZCAS-Ar 44644
- **Type status:** Paratype. **Occurrence:** recordedBy: Lihong Tu; individualCount: 3; sex: 2 males, 1 female; lifeStage: adult; occurrenceID: 898F1FA5-1B10-56DB-B083-694DF010D429; **Taxon:** order: Araneae; family: Linyphiidae; genus: Wuliphantes; **Location:** country: China; stateProvince: Sichuan; municipality: Ya'an; locality: Lushan County; **Event:** year: 2004; month: 7; day: 7; **Record Level:** institutionID: IZCAS-Ar 44645–Ar 44647

#### Description

**Male** (Holotype). Total length: 2.09. Carapace 1.05 long, 0.83 wide, yellowish-brown. Sternum 0.45 long, 0.52 wide. Clypeus 0.14 high. Chelicerae promargin with 3 teeth, retromargin with 3 teeth. Eye sizes and interdistances: AME 0.04, ALE 0.08, PME 0.08, PLE 0.09, AME-AME 0.01, PME-PME 0.02, AME-ALE 0.04, PME-PLE 0.06, coxae IV separated by 1.44 times their width. Leg measurements: I 5.09 (1.38, 0.28, 1.43, 1.27, 0.73), II 4.34 (1.20, 0.26, 1.17, 1.04, 0.67), III 3.14 (0.90, 0.22, 0.77, 0.76, 0.49), IV 4.12 (1.15, 0.23, 1.13, 1.01, 0.60). Leg formula: I-II-IV-III. TmI 0.21, TmIV absent. Tibial spine formula: 2-2-2-2. Abdomen grey, dorsum with transverse brown chevrons, ventral side grey (Fig. 7a and b).

**Figure 4.**
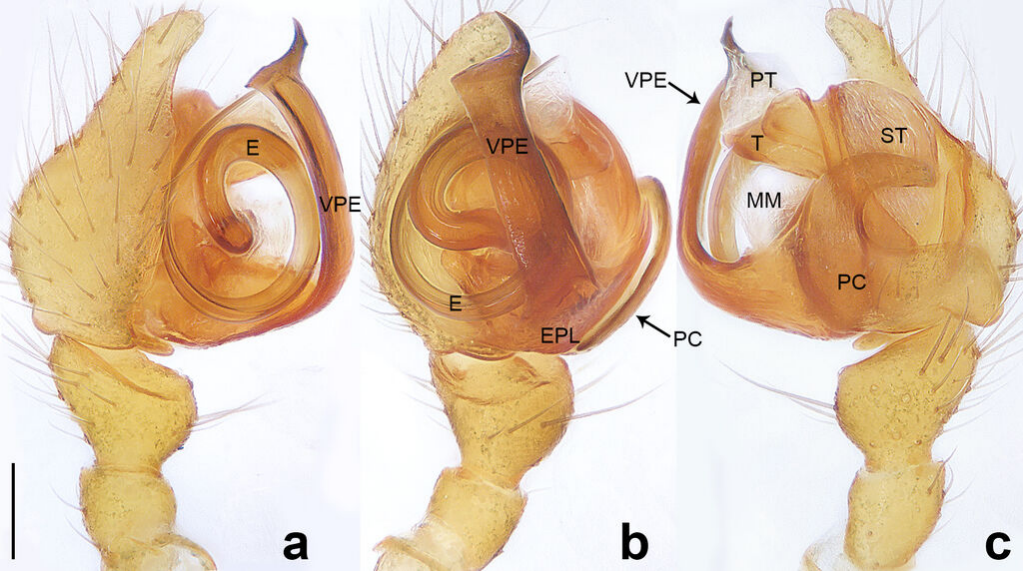
*Wuliphantesyaan* sp. nov., holotype male, left palp. **a** prolateral view; **b** ventral view; **c** retrolateral view. Scale bar: 0.10 mm (a–c).

Palp (Figs 4, 5b). Tibia with two retrolateral and one dorsal trichobothria; paracymbium U-shaped in retrolateral view, with wide base and narrow end; tegulum wider than long; protegulum conspicuous, membranous and trapezoid; embolic plate well-developed, with long ventral projection extending upwards with curved pointed tip distally; embolus spiral with one and a half coils, originating at 11 o’clock position in prolateral view.

**Female** (Paratype). Total length: 1.93. Carapace 0.76 long, 0.60 wide. Similar to male, habitus as in Fig. 7c and d. Sternum 0.47 long, 0.52 wide. Clypeus 0.12 high. Chelicerae promargin with 3 teeth, retromargin with 3 teeth. Eye sizes and interdistances: AME 0.04, ALE 0.09, PME 0.07, PLE 0.08, AME-AME 0.01, PME-PME 0.03, AME-ALE 0.03, PME-PLE 0.05, coxae IV separated by 1.50 times their width. Legs measurements: I 4.52 (1.20, 0.24, 1.24, 1.12, 0.72), II 3.92 (1.09, 0.23, 1.04, 0.93, 0.63), III 2.84 (0.84, 0.22, 0.67, 0.62, 0.49), IV 3.72 (1.03, 0.22, 0.99, 0.90, 0.58). Leg formula: I-II-IV-III. TmI 0.25, TmIV absent. Spine formula as in male.

**Figure 6.**
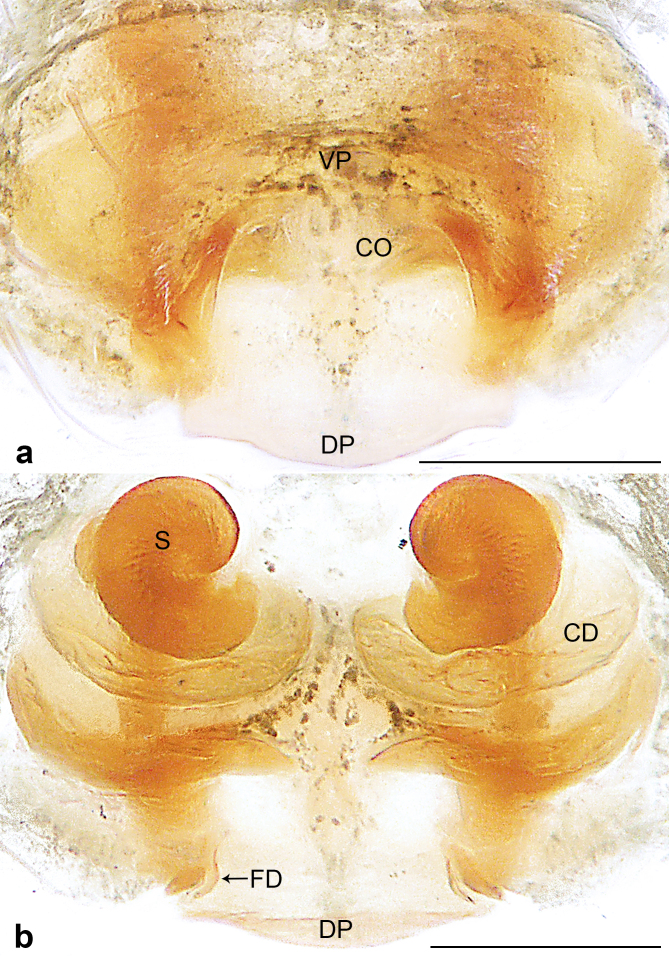
*Wuliphantesyaan* sp. nov., paratype female. **a** epigyne, ventral view; **b** vulva, dorsal view. Scale bars: 0.10 mm (a, b).

**Figure 7.**
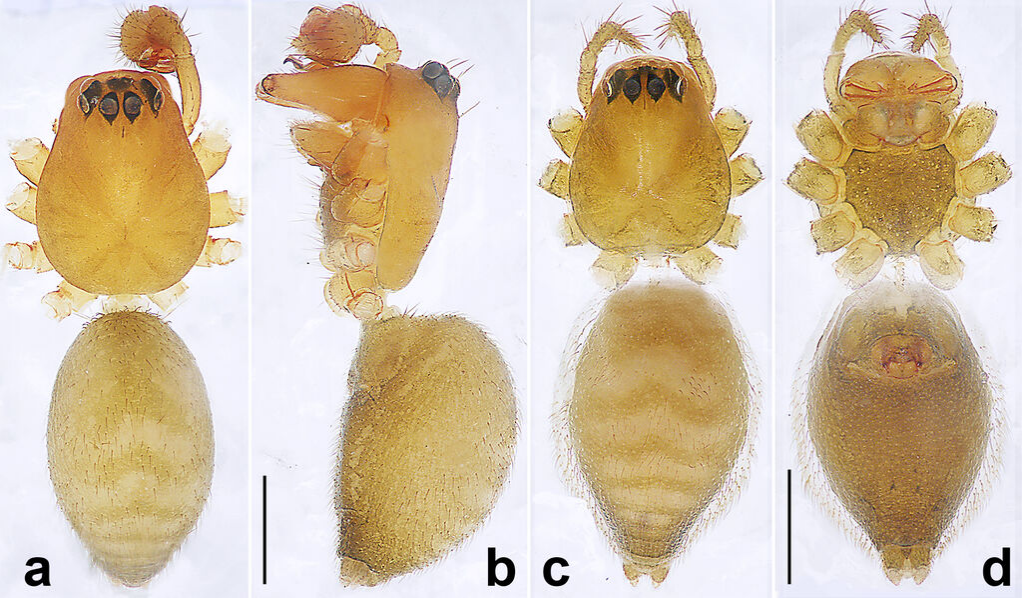
*Wuliphantesyaan* sp. nov., holotype male (a, b), paratype female (c, d). **a, c** habitus, dorsal view; **b** habitus, lateral view; **d** habitus, ventral view. Scale bars: 0.50 mm (a–d).

Epigyne (Figs 2b, 6). Ventral plate wider than long; copulatory openings apparent, near the posterior margin of ventral plate; dorsal plate inverted triangle; copulatory ducts forming two loops; spermathecae spiral; fertilisation ducts pointing antero-medially.

#### Diagnosis

The new species resembles *Wuliphantestongluensis* with similar palp and epigyne, but can be distinguished by the embolus forming 1.5 coils (Figs 4, 5b; 2.5 coils in *W.tongluensis*); by the ventral projection of embolic plate having hooked distal protrusion (Fig. 4b; sharp and narrow in *W.tongluensis*); and by the copulatory ducts only having two wide loops (Fig. 6b; five loops in *W.tongluensis*).

#### Etymology

The specific name refers to the type locality; noun in apposition.

#### Distribution

China (Sichuan, type locality).

#### Remarks

Compared with the illustrations of type species of *Microbathyphantes*: *M.palmarius* (Marples, 1955) and another species *M.spedani* (Locket,1968) provided by van Helsdingen (1985) (figs 11-17), the genital structures of the new species show significant differences that identify that the new species does not belong to genus *Microbathyphantes*. *Wuliphantesyaan* sp. nov. can be distinguished from *Microbathyphantes* by the embolus spiralling into a clock shape and approaching the length of the cymbium, but the embolus is a single coil and 1/2 length of cymbium in *Microbathyphantes*; by the base of the embolic plate completely covered by the embolus, but the embolic plate (lamella in *Microbathyphantes*) broad and uncovered by the embolus in *Microbathyphantes* (van Helsdingen 1985, figs 15 and 17); and by copulatory ducts transparent with several coiled loops, but without coiled loops in *Microbathyphantes* (van Helsdingen 1985, fig. 13). Therefore, these diagnoses suggest that the new species should belong to *Wuliphantes* rather than *Microbathyphantes*.

## Supplementary Material

XML Treatment for Wuliphantes tongluensis

XML Treatment for Wuliphantes yaan

## Figures and Tables

**Figure 2a.**
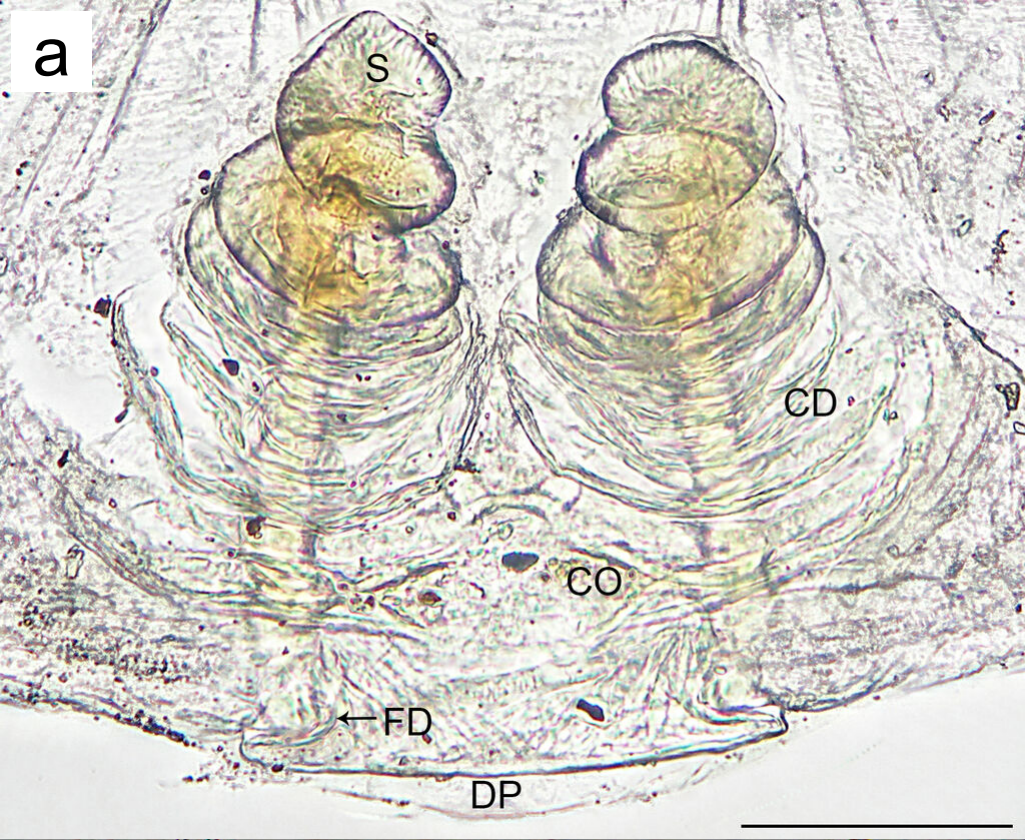
*W.tongluensis*, holotype;

**Figure 2b.**
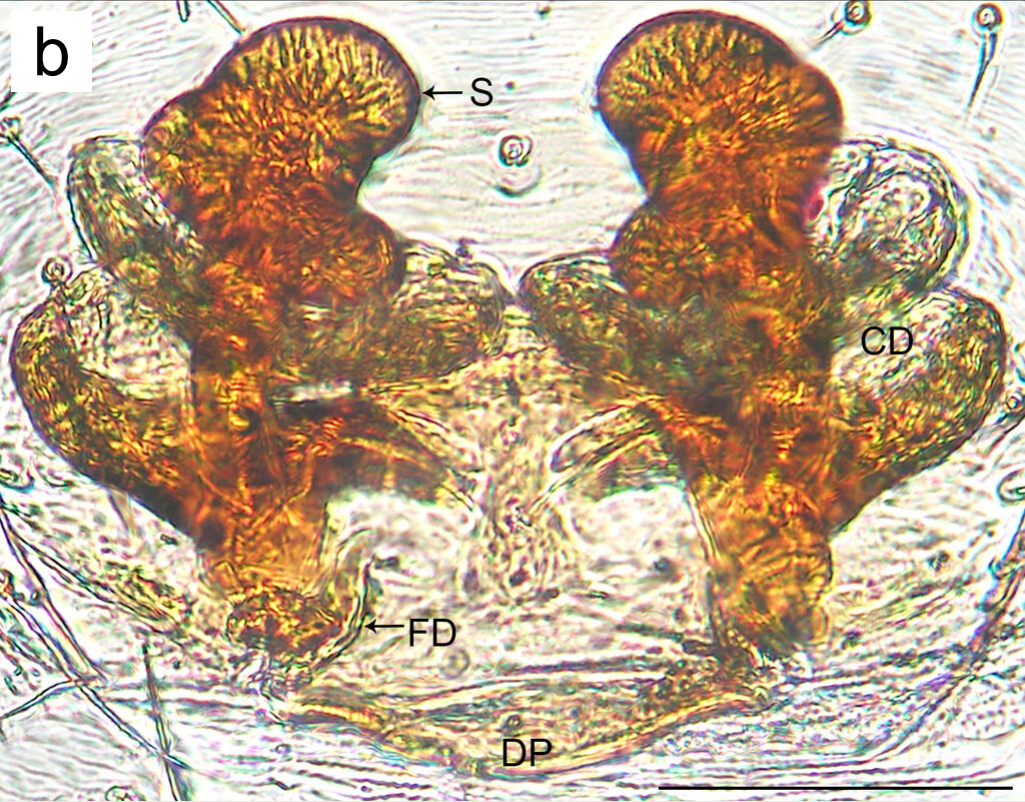
*W.yaan* sp. nov., paratype.

**Figure 5a.**
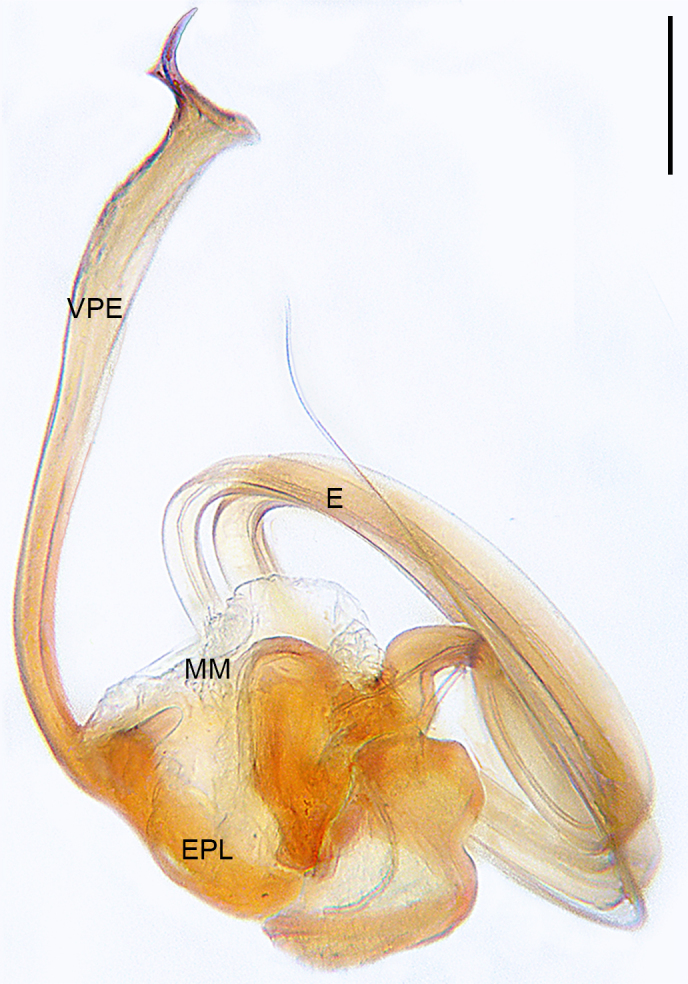
*W.tongluensis*;

**Figure 5b.**
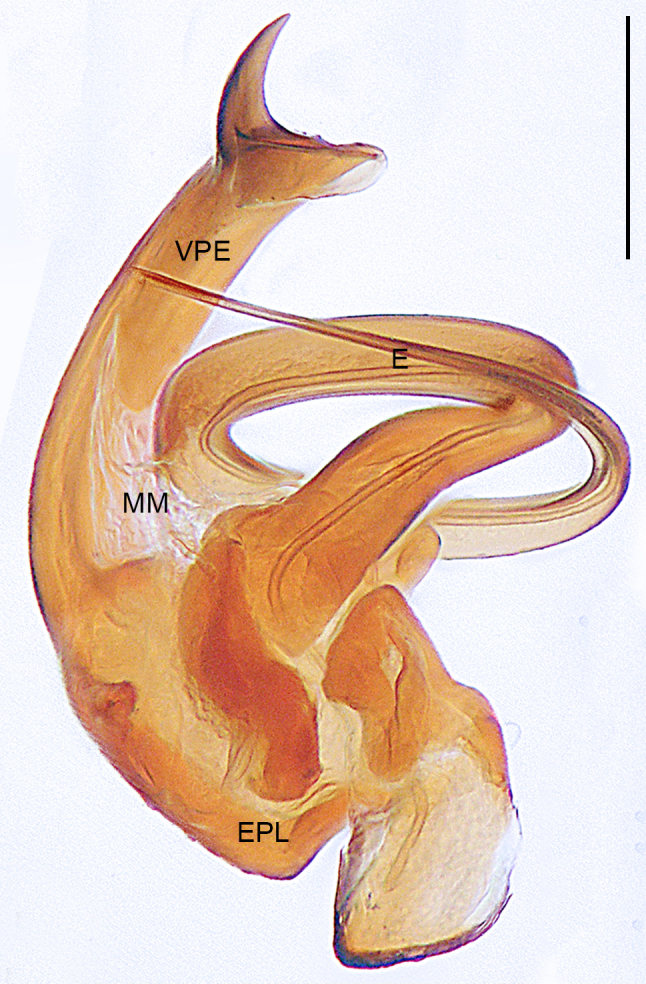
*W.yaan* sp. nov.
